# Improved Serum Alpha-Fetoprotein Levels after Iron Reduction Therapy in HCV Patients

**DOI:** 10.1155/2014/875140

**Published:** 2014-02-04

**Authors:** Hidenao Noritake, Yoshimasa Kobayashi, Yukimasa Ooba, Kensuke Kitsugi, Shin Shimoyama, Satoru Yamazaki, Takeshi Chida, Shinya Watanabe, Kazuhito Kawata, Takafumi Suda

**Affiliations:** ^1^Division of Hepatology, Department of Internal Medicine, Hamamatsu University School of Medicine, 1-20-1 Handayama, Higashi-ku, Hamamatsu, Shizuoka 431-3192, Japan; ^2^Division of Respiratology, Department of Internal Medicine, Hamamatsu University School of Medicine, 1-20-1 Handayama, Higashi-ku, Hamamatsu, Shizuoka 431-3192, Japan

## Abstract

*Background and Aims*. To examine the changes in serum alpha-fetoprotein (AFP) levels after iron reduction by therapeutic phlebotomy in chronic hepatitis C patients. *Methods*. This retrospective study included 26 chronic hepatitis C patients. The patients were developed iron depletion by repeated therapeutic phlebotomies. *Results*. Iron reduction therapy significantly reduced the median level of serum AFP from 13 to 7 ng/mL, ALT from 96 to 50 IU/L, gamma-glutamyl transpeptidase (GGT) from 55 to 28 IU/L, and ferritin from 191 to 10 ng/mL (*P* < 0.001 for each). The rate of decline in the AFP level correlated positively only with that in GGT (*r* = 0.695, *P* = 0.001), although a spurious correlation was observed between the rates of decline for AFP and ALT. The AFP level normalized (<10 ng/mL) posttreatment in eight (50%) of 16 patients who had elevated pretreatment AFP levels. Normalized post-treatment ALT and GGT levels were seen in 12% (3 of 26) and 39% (7 of 18) of the patients, respectively. Multivariate analysis identified a post-treatment GGT level of <30 IU/L as an independent factor associated with post-treatment AFP normalization (odds ratio, 21; 95% confidence interval, 1.5–293; *P* = 0.024). *Conclusions*. Iron reduction by therapeutic phlebotomy can reduce serum AFP and GGT levels in chronic hepatitis C patients.

## 1. Introduction

Estimated 170 million people worldwide are chronically infected with hepatitis C virus (HCV) [1], which is a leading cause of hepatocellular carcinoma (HCC) [2, 3]. Routine HCC screening is recommended for HCV patients, and alpha-fetoprotein (AFP) has been used widely to screen for HCC in these patients [4, 5]. However, elevated serum AFP levels are found in some patients with HCV but without HCC [6–9]; these patients are recognized as a high-risk group for HCC [10, 11].

Eradication of HCV with interferon (IFN)-based therapy, including peginterferon alpha combined with ribavirin and telaprevir or boceprevir, is the most promising therapeutic intervention for preventing the progression to HCC in HCV patients [12]. Recently, IFN-based therapy was shown to decrease serum AFP levels in HCV patients with elevated pretreatment AFP, even in IFN nonresponders [8, 13–17]. However, especially for those patients who do not respond to or are unsuitable for antiviral therapy, other therapeutic options capable of preventing the progression to HCC are urgently needed.

Iron reduction by therapeutic phlebotomy was recently shown to reduce the risk for progression to HCC and improve biochemical and histological outcomes in chronic HCV patients [18, 19]. The effect of iron reduction therapy on serum AFP levels has not been investigated fully in these patients. Therefore, this study examined the changes in serum AFP levels after iron reduction by therapeutic phlebotomy in chronic HCV patients.

## 2. Materials and Methods

### 2.1. Ethics

All patients provided written informed consent before participating in this study. The study protocol conformed to the ethics guidelines of the 1975 Declaration of Helsinki (sixth revision, 2008), as reflected in a priori approval by the institution's human research committee.

### 2.2. Patients

This study retrospectively investigated 26 consecutive chronic HCV patients (10 men, 16 women; mean age, 63 ± 18 years) with persistently elevated serum alanine aminotransferase (ALT) levels. These patients were unresponsive to (5 patients; 19%) or unsuitable (21 patients; 81%) for antiviral therapy and achieved iron depletion by repeated therapeutic phlebotomies performed in the hepatology unit of Hamamatsu University School of Medicine Hospital between January, 2000 and December, 2009. The diagnosis of chronic hepatitis *C* was based on the detection of anti-HCV antibodies and HCV-RNA in the serum. These patients met the following criteria: (1) adult > 20 years of age, (2) no anemia before therapy, as evidenced by a hemoglobin concentration >11 g/dL, (3) no hepatocellular carcinoma or other malignant disease, and (4) no habitual alcohol consumption.

### 2.3. Iron Reduction Therapy

Iron reduction therapy was administered by performing intermittent phlebotomies. All patients underwent phlebotomy with the removal of 200–400 mL of blood monthly or bimonthly until iron deficiency was achieved (defined as either a serum ferritin concentration of 10 ng/mL or a blood hemoglobin concentration of 11.0 g/dL). The subjects were required to avoid an iron-rich diet during the therapy.

### 2.4. Laboratory Tests

Routine hematological parameters, including hemoglobin concentration, platelet count, ALT, gamma-glutamyl transpeptidase (GGT), ferritin, and AFP, were measured in our clinical laboratory using standard techniques.

### 2.5. Statistical Analysis

Statistical analysis was performed using PASW Statistics, release 18.0 for Windows (SPSS, Chicago, IL, USA). All variables were tested for distribution using the Shapiro-Wilk normality test. For data not normally distributed, nonparametric statistics were used for the analyses. Quantitative pre- and posttreatment values were compared using the paired *t-*test or Wilcoxon signed-rank test. Correlation coefficients were calculated using Pearson's product-moment correlation analysis or Spearman's rank correlation analysis. Multiple linear or logistic regression analysis was performed to identify independent determinants. All tests of significance were two-sided, and *P* values <0.05 were considered to indicate statistical significance.

## 3. Results

### 3.1. Patient Characteristics

**Figure 1:**
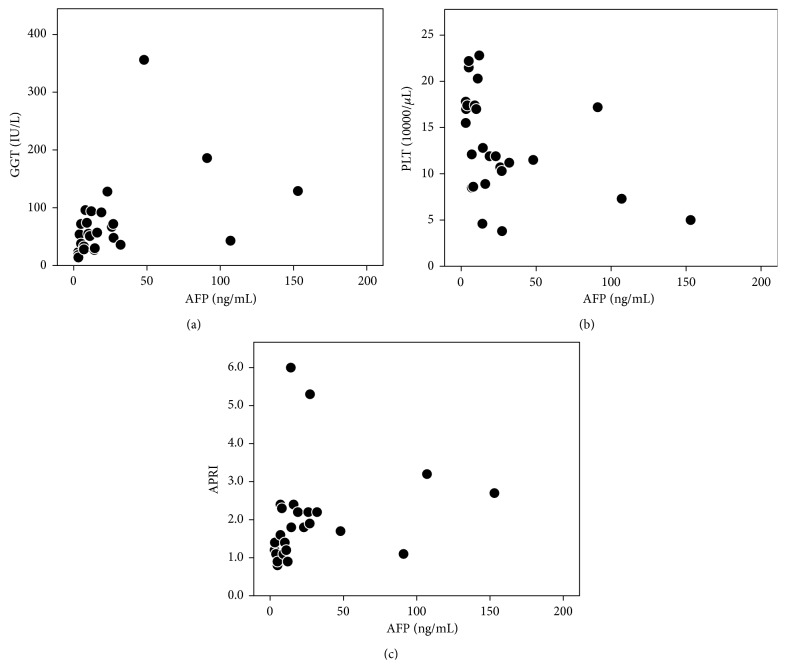
Scatter diagram showing the correlation between serum GGT level, the platelet count, APRI, and serum AFP level before phlebotomy in HCV patients. (a) The serum AFP level correlated positively with the serum GGT level (*r* = 0.542, *P* = 0.004). (b) The serum AFP level correlated negatively with the platelet count (*r* = −0.553, *P* = 0.003). (c) The serum AFP level correlated positively with APRI (*r* = 0.531, *P* = 0.005).

The demographic and clinical characteristics of the 26 patients in this study at the time of pretreatment are shown in Table 1. Fifteen (58%) patients had a platelet count ≤15 × 10^4^/*μ*L and an aspirate aminotransferase to platelet ratio index (APRI) >1.5 for the prediction of severe hepatic fibrosis [20], respectively. The median serum AFP level was 13 (range, 3–153) ng/mL, and 16 patients (62%) had elevated serum AFP (≥10 ng/mL). The serum AFP level correlated positively with the serum GGT level (*r* = 0.542, *P* = 0.004) and APRI (*r* = 0.531, *P* = 0.005) and negatively with the platelet count (*r* = −0.553, *P* = 0.003), but not with other laboratory values (Figure 1).

### 3.2. Response to Phlebotomy

All patients achieved iron depletion [median iron saturation, 6.7% (range, 2.7–19.0%); median ferritin, 10.3 (range, 4.1–43.0) ng/mL] with repeated phlebotomies. Iron depletion significantly reduced the median levels of serum AFP from 13 to 7 ng/mL, ALT from 96 to 50 IU/L, GGT from 55 to 28 IU/L, ferritin from 191 to 10 ng/mL, and hemoglobin concentration from 14.2 to 11.1 g/dL (*P* < 0.001 for each).

**Figure 2:**
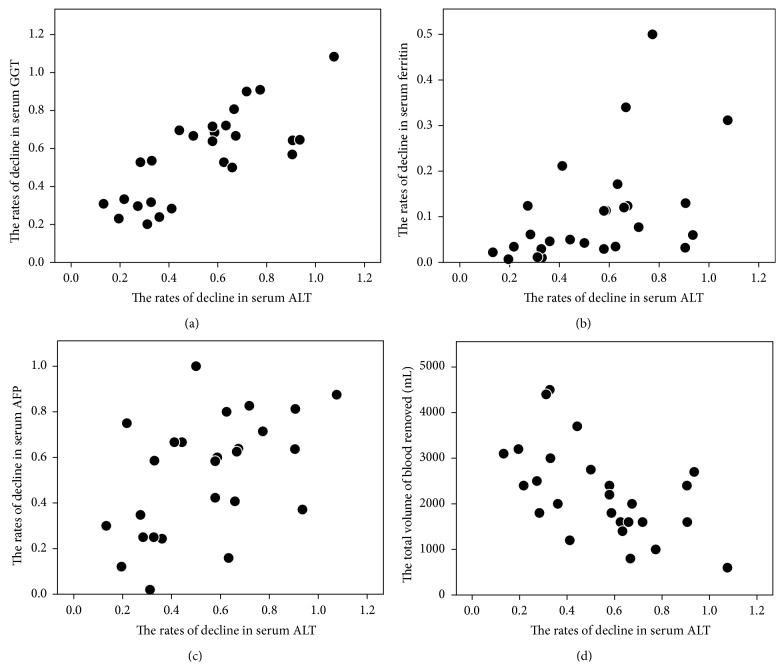
Scatter diagram showing the correlation between the rates of decline in serum GGT, ferritin, and AFP levels or the total volume of blood removed and those of serum ALT levels after phlebotomy. (a) The rates of decline in ALT levels were correlated with those in GGT (*r* = 0.723, *P* < 0.001). (b) The rates of decline in ALT levels were correlated with those in ferritin (*r* = 0.577, *P* = 0.002). (c) The rates of decline in ALT levels were correlated with those in AFP levels (*r* = 0.511, *P* = 0.008). (d) The rates of decline in ALT levels were correlated with those in the total volume of blood removed (*r* = −0.603, *P* = 0.001).

The ALT levels decreased in all patients after treatment. The rate of decline (*i*.*e*., the ratio of posttreatment to pretreatment values) in the ALT level was positively correlated with the decline in GGT (*r* = 0.723, *P* < 0.001), ferritin (*r* = 0.577, *P* = 0.002), and AFP (*r* = 0.511, *P* = 0.008) and negatively correlated with the total volume of blood removed (*r* = −0.603, *P* = 0.001), but not correlated with the change in the hemoglobin level (Figure 2). Multiple linear regression analysis identified the rate of decline in the GGT level as an independent factor associated with the rate of decline in the ALT level (*P* < 0.001). The posttreatment ALT levels normalized in three patients (12%; <30 IU/L).

**Figure 3:**
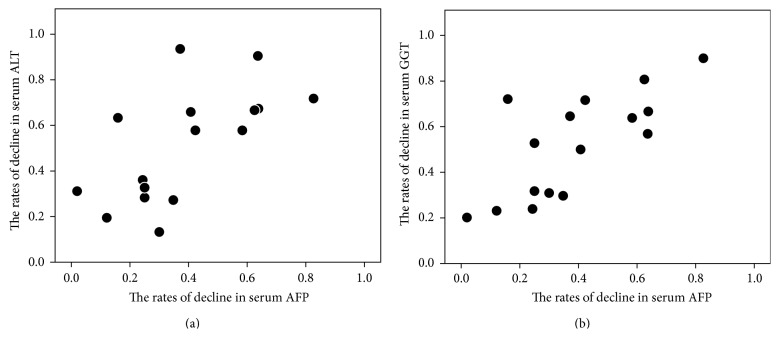
Scatter diagram showing the correlation between the rates of decline in serum ALT or GGT levels and those of serum AFP levels after phlebotomy in HCV patients with elevated pretreatment AFP. (a) The rates of decline in AFP levels were positively correlated with those in ALT (*r* = 0.685, *P* = 0.004). (b) The rates of decline in AFP levels were positively correlated with those in GGT (*r* = 0.695, *P* = 0.003).

All 16 patients with elevated pretreatment AFP (≥10 ng/mL) had decreased AFP levels after treatment. The rate of decline in the AFP level was positively correlated with the decline in ALT (*r* = 0.685, *P* = 0.004) and GGT (*r* = 0.695, *P* = 0.003), but not with decline in the hemoglobin and ferritin levels or with the total volume of blood removed (Figure 3). Multiple linear regression analysis identified the rate of decline in the GGT level as an independent factor associated with the rate of decline in the AFP level (*P* = 0.001). The posttreatment AFP levels normalized (<10 ng/mL) in eight (50%) of the 16 patients with elevated pretreatment AFP levels. The median blood volume removed by phlebotomy tended to be less for achieving posttreatment normalization of AFP than of ALT (1200 mL for AFP *versus* 2000 mL for ALT; *P* = 0.138). Multivariate analysis identified a posttreatment GGT level of <30 IU/L as an independent factor associated with posttreatment AFP normalization (odds ratio, 21; 95% confidence interval, 1.5–293; *P* = 0.024).

## 4. Discussion

Alpha-fetoprotein is a clinical serum marker for diagnosing HCC, especially in patients with chronic liver disease [4, 21]. Elevated AFP levels are also found in patients with chronic viral hepatitis and cirrhosis without HCC. The incidence of elevated AFP levels in HCV patients without HCC ranges from 10 to 43% [8, 9, 22, 23]. In the present study, 62% of the subjects had elevated pretreatment AFP levels. This higher incidence might be explained by a different patient population, sample size, or definition of serum AFP elevation. An elevated AFP in HCV patients without HCC is reportedly associated with gender, age, ethnicity, platelet count, transaminase levels, viral genotype, and liver histology [6–8]. Although the predictors for an elevated AFP in HCV patients are diverse, an advanced fibrosis stage is the most common predictor, and this finding was consistent in most previous studies [6–8]. We found that a higher GGT level, lower platelet count, and higher APRI were associated with pretreatment AFP elevation and these factors are known to predict advanced hepatic fibrosis [20, 24–27]. The association observed in this study between AFP, GGT, platelet count, and APRI may be attributable to the increased expression of hepatic progenitor cells (HPCs) in advanced hepatic fibrosis. AFP and GGT are highly expressed in HPCs [28] and are important in hepatocyte regeneration with impaired replication of mature hepatocytes in most liver diseases [29–31]. HPC expansion is associated with the severity of hepatic fibrosis in chronic HCV infection [29, 32, 33]. Our recent preliminary study found that the serum AFP and GGT levels were positively correlated with the degree of HPC activation in HCV patients with elevated serum AFP levels (≥10 ng/mL) without HCC (personal communication, 2012).

Previous studies have suggested that an elevated AFP level is an independent risk factor for developing HCC [10, 11]. Therapeutic intervention to reduce serum AFP levels may prevent hepatocarcinogenesis in chronic HCV infection. Several studies have reported that IFN therapy can reduce AFP levels and the likelihood of hepatocarcinogenesis [8, 13–17]. Nevertheless, other therapeutic options capable of preventing the progression to HCC are urgently needed for HCV patients who do not respond to or are unsuitable for IFN therapy and those who cannot afford the therapy. Iron reduction therapy by phlebotomy alone or in combination with a low-iron diet is an inexpensive treatment with few side effects and may reduce the risk of hepatocarcinogenesis associated with HCV [18].

A few studies have shown that phlebotomy can reduce serum AFP levels in patients with chronic hepatitis C [34–36]. However, the full effect of phlebotomy on serum AFP levels in HCV patients is not clear. In the present study, all HCV patients with elevated pretreatment AFP had decreased AFP levels after treatment. Furthermore, normalization of the posttreatment AFP level (<10 ng/mL) was observed even in patients without normalization of posttreatment ALT levels. Finally, the volume of blood removed by phlebotomy was less for normalizing the posttreatment level of AFP than that of ALT. These observations suggest that serum AFP responds better to phlebotomy than serum ALT. In addition, this study found a strong correlation between the decline in the AFP and GGT levels with phlebotomy. This may reflect the amelioration of enhanced HPC accumulation and upregulated AFP and GGT expression by the treatment. Given that HPC transformation has been proposed to lead to HCC [37], a reduced level of AFP, a marker of HPCs, may reduce the likelihood of HCC development in HCV patients treated with phlebotomy.

The mechanisms responsible for AFP reduction by phlebotomy remain unclear. In chronic HCV infection, iron-mediated oxidative stress contributes to hepatic injury [38] and therapeutic iron depletion decreases hepatic oxidative stress [39]. GGT plays an important role in antioxidant defenses by participating in the metabolism of glutathione [40] and is upregulated after exposure to oxidative stress [41]. Thus, the decrease in GGT observed in this study most likely reflects the improvement in hepatic iron-mediated oxidative stress with phlebotomy. Oxidative stress also inhibits the replication of mature hepatocytes, resulting in the activation of HPCs [42, 43]. HPC accumulation is observed in diseased livers with enhanced hepatic oxidative stress, including livers with chronic hepatitis C [44, 45]. Therefore, the amelioration of enhanced hepatic iron-mediated oxidative stress and the related HPC expansion is likely to contribute to the decrease of AFP with iron reduction by phlebotomy.

With the development of potent direct-acting antiviral agents combinations, IFN-free therapy is likely to be approved in the near feature [46]. However, the portion of HCV patients is not eligible for this new therapy because of drug toxicity, drug-drug interactions, the induction of long-term viral resistance, and medical cost. Therefore, iron reduction therapy by phlebotomy remains useful for these patients.

Further study is needed to determine if iron reduction therapy can reduce the risk of the development of HCC in HCV patients with elevated AFP. Similar intervention may contribute to the amelioration of increased risk for various iron-related cancers including HCC and colorectal cancer in patients with genetic hemochromatosis [47–49], considering that genetic modifications and continual activation of the signalling pathways of cell proliferation by iron-mediated reactive oxygen species promote carcinogenesis synergistically [50, 51].

In conclusion, iron reduction by therapeutic phlebotomy can reduce the serum AFP and GGT levels in HCV patients. This is probably mediated by the amelioration of enhanced hepatic iron-mediated oxidative stress and the resultant HPC expansion. This therapy may reduce the risk for progression to HCC in chronic HCV infection.

## Figures and Tables

**Table 1:** Clinical findings before and after phlebotomy in 26 HCV patients.

Variables	Pretreatment	Posttreatment	*P* value
Age (yr) mean ± S.D.	63 ± 18		
Gender (male/female)	10/16		
Previous antiviral therapy (naïve/treatment experienced)	(21/5)		
AST to platelet ratio index (APRI)	1.8 (0.8–6.0)		
Total volume of blood removed (L) mean ± S.D.		2.2 ± 10	
Treatment period (month) mean ± S.D.		8.3 ± 4.5	
Hemoglobin (g/dL)	14.2 (12.3–15.7)	11.1 (8.7–13.0)	<0.001
Platelet (×10^4^/*μ*L)	12.0 (3.8–22.8)	14.8 (5.6–31.9)	<0.001
Total bilirubin (mg/dL)	0.8 (0.4–1.6)	0.7 (0.2–1.1)	<0.001
ALT (IU/L)	96 (32–364)	50 (27–96)	<0.001
GGT (IU/L)	55 (14–356)	28 (9–113)	<0.001
Ferritin (ng/mL)	191 (18–1010)	10 (4–43)	<0.001
Iron saturation (%)	6.7 (2.7–19.0)	10.3 (4.1–43.0)	<0.001
AFP (ng/mL)	13 (3–153)	7 (2–17)	<0.001

Unless otherwise indicated, values represent median (range).

AST: aspartate aminotransferase, ALT: alanine aminotransferase, GGT: gamma-glutamyl transpeptidase, and AFP: alpha-fetoprotein.

## References

[1] World Health Organization Hepatitis C. http://www.who.int/csr/disease/hepatitis/whocdscsrlyo2003/en/index.html.

[2] Hoofnagle J. H. (2002). Course and outcome of hepatitis C. *Hepatology*.

[3] Seeff L. B. (2002). Natural history of chronic hepatitis C. *Hepatology*.

[4] Sato Y., Nakata K., Kato Y. (1993). Early recognition of hepatocellular carcinoma based on altered profiles of alpha-fetoprotein. *The New England Journal of Medicine*.

[5] Johnson P. J. (2001). The role of serum alpha-fetoprotein estimation in the diagnosis and management of hepatocellular carcinoma. *Clinics in Liver Disease*.

[6] Hu K.-Q., Kyulo N. L., Lim N., Elhazin B., Hillebrand D. J., Bock T. (2004). Clinical significance of elevated alpha-fetoprotein (AFP) in patients with chronic hepatitis C, but not hepatocellular carcinoma. *The American Journal of Gastroenterology*.

[7] Chu C.-W., Hwang S.-J., Luo J.-C. (2001). Clinical, virologic, and pathologic significance of elevated serum alpha-fetoprotein levels in patients with chronic hepatitis C. *Journal of Clinical Gastroenterology*.

[8] Di Bisceglie A. M., Sterling R. K., Chung R. T. (2005). Serum alpha-fetoprotein levels in patients with advanced hepatitis C: results from the HALT-C Trial. *Journal of Hepatology*.

[9] Fried M. W., Shiffman M. L., Rajender Reddy K. (2002). Peginterferon alfa-2a plus ribavirin for chronic hepatitis C virus infection. *The New England Journal of Medicine*.

[10] Ikeda K., Arase Y., Saitoh S. (2006). Prediction model of hepatocarcinogenesis for patients with hepatitis C virus-related cirrhosis: validation with internal and external cohorts. *Journal of Hepatology*.

[11] Akuta N., Suzuki F., Kawamura Y. (2008). Substitution of amino acid 70 in the hepatitis C virus core region of genotype 1b is an important predictor of elevated alpha-fetoprotein in patients without hepatocellular carcinoma. *Journal of Medical Virology*.

[12] Cardoso A.-C., Moucari R., Figueiredo-Mendes C. (2010). Impact of peginterferon and ribavirin therapy on hepatocellular carcinoma: incidence and survival in hepatitis C patients with advanced fibrosis. *Journal of Hepatology*.

[13] Murashima S., Tanaka M., Haramaki M. (2006). A decrease in AFP level related to administration of interferon in patients with chronic hepatitis C and a high level of AFP. *Digestive Diseases and Sciences*.

[14] Arase Y., Ikeda K., Suzuki F. (2007). Prolonged-interferon therapy reduces hepatocarcinogenesis in aged-patients with chronic hepatitis C. *Journal of Medical Virology*.

[15] Chen T.-M., Huang P.-T., Tsai M.-H. (2007). Predictors of alpha-fetoprotein elevation in patients with chronic hepatitis C, but not hepatocellular carcinoma, and its normalization after pegylated interferon alfa 2a-ribavirin combination therapy. *Journal of Gastroenterology and Hepatology*.

[16] Akuta N., Suzuki F., Kawamura Y. (2008). Efficacy of low-dose intermittent interferon-alpha monotherapy in patients infected with hepatitis C virus genotype 1b who were predicted or failed to respond to pegylated interferon plus ribavirin combination therapy. *Journal of Medical Virology*.

[17] Yu M.-L., Lin S.-M., Chuang W.-L. (2006). A sustained virological response to interferon or interferon/ribavirin reduces hepatocellular carcinoma and improves survival in chronic hepatitis C: a nationwide, multicentre study in Taiwan. *Antiviral Therapy*.

[18] Kato J., Miyanishi K., Kobune M. (2007). Long-term phlebotomy with low-iron diet therapy lowers risk of development of hepatocellular carcinoma from chronic hepatitis C. *Journal of Gastroenterology*.

[19] Franchini M., Targher G., Capra F., Montagnana M., Lippi G. (2008). The effect of iron depletion on chronic hepatitis C virus infection. *Hepatology International*.

[20] Wai C.-T., Greenson J. K., Fontana R. J. (2003). A simple noninvasive index can predict both significant fibrosis and cirrhosis in patients with chronic hepatitis C. *Hepatology*.

[21] Johnson P. J. (2001). The role of serum alpha-fetoprotein estimation in the diagnosis and management of hepatocellular carcinoma. *Clinics in Liver Disease*.

[22] Hu K.-Q., Kyulo N. L., Lim N., Elhazin B., Hillebrand D. J., Bock T. (2004). Clinical significance of elevated alpha-fetoprotein (AFP) in patients with chronic hepatitis C, but not hepatocellular carcinoma. *The American Journal of Gastroenterology*.

[23] Chu C.-W., Hwang S.-J., Luo J.-C. (2001). Clinical, virologic, and pathologic significance of elevated serum alpha-fetoprotein levels in patients with chronic hepatitis C. *Journal of Clinical Gastroenterology*.

[24] Taniguchi H., Iwasaki Y., Fujiwara A. (2006). Long-term monitoring of platelet count, as a non-invasive marker of hepatic fibrosis progression and/or regression in patients with chronic hepatitis C after interferon therapy. *Journal of Gastroenterology and Hepatology*.

[25] Ono E., Shiratori Y., Okudaira T. (1999). Platelet count reflects stage of chronic hepatitis C. *Hepatology Research*.

[26] Wai C.-T., Greenson J. K., Fontana R. J. (2003). A simple noninvasive index can predict both significant fibrosis and cirrhosis in patients with chronic hepatitis C. *Hepatology*.

[27] Lu S.-N., Wang J.-H., Liu S.-L. (2006). Thrombocytopenia as a surrogate for cirrhosis and a marker for the identification of patients at high-risk for hepatocellular carcinoma. *Cancer*.

[28] Dollé L., Best J., Mei J. (2010). The quest for liver progenitor cells: a practical point of view. *Journal of Hepatology*.

[29] Lowes K. N., Brennan B. A., Yeoh G. C., Olynyk J. K. (1999). Oval cell numbers in human chronic liver diseases are directly related to disease severity. *The American Journal of Pathology*.

[30] Libbrecht L., Desmet V., Van Damme B. (2000). Deep intralobular extension of human hepatic “progenitor cells” correlates with parenchymal inflammation in chronic viral hepatitis: can “progenitor cells” migrate?. *Journal of Pathology*.

[31] Eleazar J. A., Memeo L., Jhang J. S. (2004). Progenitor cell expansion: an important source of hepatocyte regeneration in chronic hepatitis. *Journal of Hepatology*.

[32] Fotiadu A., Tzioufa V., Vrettou E., Koufogiannis D., Papadimitriou C. S., Hytiroglou P. (2004). Progenitor cell activation in chronic viral hepatitis. *Liver International*.

[33] Clouston A. D., Powell E. E., Walsh M. J., Richardson M. M., Demetris A. J., Jonsson J. R. (2005). Fibrosis correlates with a ductular reaction in hepatitis C: roles of impaired replication, progenitor cells and steatosis. *Hepatology*.

[34] Sartori M., Andorno S., Rigamonti C., Baldoroni R. (2001). Chronic hepatitis C treated with phlebotomy alone: biochemical and histological outcome. *Digestive and Liver Disease*.

[35] Tanaka N., Horiuchi A., Yamaura T., Komatsu M., Tanaka E., Kiyosawa K. (2007). Efficacy and safety of 6-month iron reduction therapy in patients with hepatitis C virus-related cirrhosis: a pilot study. *Journal of Gastroenterology*.

[36] Yoshida Y., Imai Y., Sawai Y. (2010). Hydroxyoctadecadienoic acid as a potential biomarker for oxidative stress in patients with chronic hepatitis C. *Journal of Gastroenterology and Hepatology*.

[37] Libbrecht L. (2006). Hepatic progenitor cells in human liver tumor development. *World Journal of Gastroenterology*.

[38] Isom H. C., McDevitt E. I., Moon M. S. (2009). Elevated hepatic iron: a confounding factor in chronic hepatitis C. *Biochimica et Biophysica Acta*.

[39] Franchini M., Targher G., Capra F., Montagnana M., Lippi G. (2008). The effect of iron depletion on chronic hepatitis C virus infection. *Hepatology International*.

[40] Zhang H., Forman H. J. (2009). Redox regulation of *γ*-glutamyl transpeptidase. *The American Journal of Respiratory Cell and Molecular Biology*.

[41] Ravuri C., Svineng G., Pankiv S., Huseby N.-E. (2011). Endogenous production of reactive oxygen species by the NADPH oxidase complexes is a determinant of *γ*-glutamyltransferase expression. *Free Radical Research*.

[42] Ohlson L. C. E., Koroxenidou L., Hällström I. P. (1998). Inhibition of in vivo rat liver regeneration by 2-acetylaminofluorene affects the regulation of cell cycle-related proteins. *Hepatology*.

[43] Lindeman B., Skarpen E., Oksvold M. P. (2000). The carcinogen 2-acetylaminofluorene inhibits activation and nuclear accumulation of cyclin-dependent kinase 2 in growth-induced rat liver. *Molecular Carcinogenesis*.

[44] Schroeijers A. B., Scheffer G. L., Flens M. J. (1998). Immunohistochemical detection of the human major vault protein LRP with two monoclonal antibodies in formalin-fixed, paraffin-embedded tissues. *The American Journal of Pathology*.

[45] Roskams T., Yang S. Q., Koteish A. (2003). Oxidative stress and oval cell accumulation in mice and humans with alcoholic and nonalcoholic fatty liver disease. *The American Journal of Pathology*.

[46] Liang T. J., Ghany M. G. (2013). Current and future therapies for hepatitis C virus infection. *The New England Journal of Medicine*.

[47] Chua A. C. G., Klopcic B., Lawrance I. C., Olynyk J. K., Trinder D. (2010). Iron: an emerging factor in colorectal carcinogenesis. *World Journal of Gastroenterology*.

[48] Tirnitz-Parker J. E., Glanfield A., Olynyk J. K. (2013). Iron and hepatic carcinogenesis. *Critical Reviews in Oncogenesis*.

[49] Asberg A., Thorstensen K., Irgens W. (2013). Cancer risk in HFE C282Y homozygotes: results from the HUNT 2 study. *Scandinavian Journal of Gastroenterology*.

[50] Galaris D., Skiada V., Barbouti A. (2008). Redox signaling and cancer: the role of “labile” iron. *Cancer Letters*.

[51] Toyokuni S. (1995). Persistent oxidative stress in cancer. *FEBS Letters*.

